# Regional homogeneity alterations in multifrequency bands in patients with basal ganglia stroke: A resting-state functional magnetic resonance imaging study

**DOI:** 10.3389/fnagi.2022.938646

**Published:** 2022-08-11

**Authors:** Qianqian Li, Su Hu, Yingmin Mo, Hao Chen, Chaoguo Meng, Linlin Zhan, Mengting Li, Xuemei Quan, Yanyan Gao, Lulu Cheng, Zeqi Hao, Xize Jia, Zhijian Liang

**Affiliations:** ^1^Department of Neurology, The First Affiliated Hospital of Guangxi Medical University, Nanning, China; ^2^School of Teacher Education, Zhejiang Normal University, Jinhua, China; ^3^The Cadre Ward in Department of Neurology, The People’s Hospital of Guangxi Zhuang Autonomous Region, Nanning, China; ^4^Faculty of Western Languages, Heilongjiang University, Harbin, China; ^5^Key Laboratory of Intelligent Education Technology and Application of Zhejiang Province, Zhejiang Normal University, Jinhua, China; ^6^Department of Neurology, The People’s Hospital of Guangxi Zhuang Autonomous Region, Nanning, China; ^7^School of Foreign Studies, China University of Petroleum (East China), Qingdao, China; ^8^Shanghai Center for Research in English Language Education, Shanghai International Studies University, Shanghai, China

**Keywords:** resting-state fMRI, regional homogeneity (ReHo), frequency-specific, ischemic stroke, basal ganglia

## Abstract

**Objective:**

The aim of this study was to investigate the spontaneous regional neural activity abnormalities in patients with acute basal ganglia ischemic stroke (BGIS) using a multifrequency bands regional homogeneity (ReHo) method and to explore whether the alteration of ReHo values was associated with clinical characteristics.

**Methods:**

In this study, 34 patients with acute BGIS and 44 healthy controls (HCs) were recruited. All participants were examined by resting-state functional magnetic resonance imaging (rs-fMRI). The ReHo method was used to detect the alterations of spontaneous neural activities in patients with acute BGIS. A two-sample *t*-test comparison was performed to compare the ReHo value between the two groups, and a Pearson correlation analysis was conducted to assess the relationship between the regional neural activity abnormalities and clinical characteristics.

**Results:**

Compared with the HCs, the patients with acute BGIS showed increased ReHo in the left caudate and subregions such as the right caudate and left putamen in conventional frequency bands. In the slow-5 frequency band, patients with BGIS showed decreased ReHo in the left medial cingulum of BGIS compared to the HCs and other subregions such as bilateral caudate and left putamen. No brain regions with ReHo alterations were found in the slow-4 frequency band. Moreover, we found that the ReHo value of left caudate was positively correlated with the NIHSS score.

**Conclusion:**

Our findings revealed the alterations of ReHo in patients with acute BGIS in a specific frequency band and provided a new insight into the pathogenesis mechanism of BGIS. This study demonstrated the frequency-specific characteristics of ReHo in patients with acute BGIS, which may have a positive effect on the future neuroimaging studies.

## Introduction

As one of the major causes of mortality and disability, stroke has been regarded as the third cause of death in China in recent years (Wang et al., 2017; Zhou et al., 2019). It is estimated that 80% of strokes are ischemic strokes (Rothwell, 2012). Motor dysfunction usually leads to disability in stroke survivors. Furthermore, 75% of stroke survivors had motor dysfunction, which usually results in lifelong disability (Krishnamurthi et al., 2018). The severity of motor dysfunction in patients with ischemic stroke is related to the site of the stroke lesion (De Haan et al., 1995; Shelton and Reding, 2001). Most patients with stroke have coexisting lesions in different locations, so the most previous studies did not limit their range of sites to a single lesion location (Wu et al., 2015). The basal ganglia (BG) region is a common site for strokes and engages in the process of motor execution and motor control (Florio et al., 2018). Patients with acute basal ganglia ischemic stroke (BGIS) are more likely to suffer from motor dysfunction, which may hugely affect the patient’s quality of life (Bai et al., 2014). From this perspective, investigation of the motor dysfunction neural mechanism of BGIS is of great importance and may provide new insights for the rehabilitation of patients with BGIS in the future studies.

Previous task-based functional magnetic resonance imaging (task-based fMRI) studies have demonstrated that alterations of neuronal activity in certain regions of the brain cortex are associated with stroke-related motor dysfunction (Du et al., 2018; Hannanu et al., 2020). However, the practice and application of such research are difficult because task-based fMRI requires patients to perform specific tasks during the examination, which is hard for stroke patients with severe motor dysfunction. Recently, resting-state functional magnetic resonance imaging (rs-fMRI) has gained much more attention because it only requires patients to maintain a relaxed and awake state so that it can detect spontaneous neural activity by examining the fluctuations in blood oxygenation (Wang et al., 2008; Xie et al., 2008). Therefore, compared with task-based fMRI, rs-fMRI is a more suitable and promising technology to study the intrinsic neural activities of stroke (Zhu et al., 2015; Gao et al., 2021). A study demonstrated that rs-fMRI could be used to assess the functional reorganization of the injured brain in patients with stroke (Cui et al., 2022). Moreover, Boukrina et al. (2022) used rs-fMRI to explore the relationship between arousal network activity and delirium after stroke. rs-fMRI has been widely used in the study of stroke.

The regional homogeneity (ReHo) method is a kind of analytical method for rs-fMRI, which was first proposed by Zang et al. (2004). Based on the calculation of Kendall coefficients, ReHo was designed to measure the similarity of the time series of a given voxel with its nearest neighbors and to detect the subtly altered synchronization of neuronal activity in the specific brain region. ReHo could facilitate clinicians to observe changes in neuronal activity in different diseases. Moreover, ReHo has several advantages such as parametric-setting-free, unnecessary requirement on *a priori* knowledge of the structure or function of the brain, and being more robust against noise in the data (Zuo et al., 2013). This method has been widely used to explore the local neural activities in a variety of neurological diseases, including epilepsy (Zhao et al., 2020), Alzheimer’s disease (Zhang et al., 2012), and stroke (Wu et al., 2015). A previous study found increased ReHo in the inferior parietal lobule (IPL) and middle frontal gyrus among stroke patients with hand motor dysfunction in the conventional frequency band, and this finding may have physiological underpinnings (Yin et al., 2013). Therefore, we hypothesized that ReHo is advantageous for studying changes in neural activity of BGIS.

Buzsáki and Draguhn (2004) found that brain oscillations of different frequency bands were correlated with specific neural activities and proposed a classification of different frequency bands. The low-frequency fluctuation of the conventional band (0.01–0.08 Hz) was associated with intrinsic neural activity and has the physiological meaning (Biswal et al., 1995). The slow-4 band (0.027–0.073 Hz) was more advantageous in reflecting the signals of gray neural activities (Zuo et al., 2010). The slow-5 band (0.01–0.027 Hz) was sensitive enough to reflect neural activity in a wide range of cerebral cortical regions (Zhu et al., 2015). However, most of the previous ReHo studies about stroke have focused on the conventional frequency bands (Chen et al., 2020; Gao et al., 2021). Recently, Zhu et al. (2015) found that low-frequency oscillations (LFOs) in patients with stroke would be influenced by different frequency bands. The LFOs of patients with subcortical stroke had a unique rs-fMRI frequency-dependent pattern (Zhao et al., 2018). Therefore, the frequency-dependent characteristic should be considered in the studies of stroke. However, the features of frequency-dependent in acute BGIS are still unclear. We performed a ReHo analysis in three different frequency bands (conventional frequency band, slow-4 frequency band, and slow-5 frequency band) to reveal the neural activity alterations and the characteristics of frequency-dependent in patients with acute BGIS.

In this study, we utilized ReHo to investigate the alterations of synchronized neural activity in patients with acute BGIS in three different frequency bands. The goals of our study were to explore the patterns of BGIS neuronal activities in different frequency bands and the correlations between the ReHo value of certain brain regions and clinical characteristics. Therefore, we hypothesized that the ReHo could observe the abnormal neural activities of BGIS and that the alteration of ReHo of BGIS was frequency-specific.

## Materials and methods

### Participants

This study was approved by the Ethics Committee of the First Affiliated Hospital of Guangxi Medical University. All participants signed informed consent before the study. From May 2019 to December 2020, 43 patients with BGIS and 47 age-matched HCs with no history of neurological disease were recruited. The inclusion criteria were as follows: (1) age between 30 and 75 years; (2) first-ever single lesion at BG confirmed by magnetic resonance imaging (MRI); (3) ischemic stroke accompanied with motor dysfunction onset within 10 days; (4) no contraindication to MRI scan; and (5) right-handed before stroke according to the Edinburgh Hiring Questionnaire (Oldfield, 1971). The exclusion criteria were as follows: (1) stroke lesion located in another brain region rather than the BG; (2) history of psychiatric or other neurological diseases; (3) the modified Fazekas score > 1 (Fazekas et al., 1987); (4) severe dysfunctions of auditory, vision, speech, cognition, or other physical diseases; and (5) lack of structural MRI images or poor standardization effect and any head motions more than 3.0 mm of any direction of x, y, or z or greater than 3° of rotation at any direction during the scanning.

A total of nine patients with BGIS were excluded from the study due to excessive head motion (*n* = 2), MRI image data missing (*n* = 1), poor image data quality (*n* = 1), and incomplete scanning of the cerebellum (*n* = 5), and three HCs were excluded due to incomplete scanning of the cerebellum (*n* = 1), excessive head motion (*n* = 1), and poor image data quality (*n* = 1), leaving 34 patients with BGIS and 44 HCs in the final analysis.

### Neurological assessments

All the neurological assessments of patients with BGIS were completed by neurologists. The National Institute of Health Stroke Score (NIHSS) was used to assess functional impairment caused by BGIS. NIHSS score included 13 examination items and ranged from 0 to 42, with higher scores indicating a more severe neurological deficit caused by stroke. Barthel Index (BI) was used to assess functional status and ability to perform daily life activities in BGIS. BI included the following 10 parts of scales: feeding, bathing, grooming, dressing, bowels and bladder, toilet use, transfers, mobility, and stairs climbing, and the score ranged from 0 (total dependence) to 100 (complete independence) (Mahoney and Barthel, 1965). Fugl-Meyer assessment (FMA) scale was used to assess motor function in BGIS, and it contained two components that can evaluate the movements of the upper and lower limbs separately (Fugl-Meyer et al., 1975). The score of FMA ranged from 0 to 100 and the upper and lower limb section accounts for 66 points and 34 points, and higher scores indicated a better limb motor function. We used FMA-up and FMA-low to represent the assessment of the upper and lower limbs, respectively.

### Magnetic resonance imaging data acquisition

All the MRI data were acquired from a 3.0T MRI scanner (SIEMENS MAGNETOM Prisma, Germany) at the department of radiology of the First Affiliated Hospital of Guangxi Medical University. During the fMRI scans, participants were instructed to remain awake, relaxed with their eyes closed, and kept motionless as much as possible. The standard scanning protocol was strictly obeyed using a 64-pass phased-array head coil. The parameters of MRI scanning sequences are as follows: (1) T1-weighted images were collected using a 3D BRAVO sequence with the following parameters: repetition time (TR) = 2,300 ms, echo time (TE) = 2.98 ms, inversion time = 900 ms, slice thickness = 1 mm, voxel size = 1 mm × 1 mm × 1 mm, interval = 0 mm, and field of view (FOV) = 256 mm × 256 mm; (2) rs-fMRI data were obtained using the echo-planar imaging (EPI) sequence with parameters as follows: TR/TE = 2,000/35 ms, slice thickness = 3 mm, voxel = 2.6 mm × 2.6 mm × 3 mm, slice number = 40, matrix = 64 × 64, field of view (FOV) = 240 mm × 240 mm, and flip angle (FA) = 90°.

### Data preprocessing

All the processing of rs-fMRI data was performed on the MATLAB R2017b working platform.^1^ rs-fMRI data preprocessing and statistical analyses were using the Resting-State fMRI Data Analysis Toolkit plus V1.24 (RESTplus V1.24)^2^ and Statistical Parametric Mapping (SPM 12).^3^ Statistical analyses and multiple comparison corrections were performed using Data Processing & Analysis for Brain Imaging (DPABI) V5.1.^4^ Data preprocessing steps include (1) discarding the first 10 time points to make the participants adapt to the environment and to make the machine achieve a stable state; (2) slice-timing correction to reduce the difference caused by scanning time; (3) head motions correction was conducted to correct every image at the same position (Chao-Gan and Yu-Feng, 2010); (4) the spatial normalization of functional images performed in the Montreal Neurological Institute (MNI) space by the deformation fields derived from tissue segmentation of the structural images (resampling voxel size to 3 mm × 3 mm × 3 mm); (5) eliminating linear trend caused by a machine whose temperature is heating up; (6) nuisance regression was completed by regressing out covariables like white matter signal noise, cerebrospinal fluid signal noise (CSF), and head movement effect (using Friston-24 model) to further eliminate the influence of head motion (Friston et al., 1996) and non-neuronal BOLD fluctuations (Fox et al., 2005); and (7) filtering the data *via* the conventional frequency band, slow-5 frequency band, and slow-4 frequency bands.

### Regional homogeneity calculation

Calculated by REST plus, the ReHo value across the whole brain was conducted by the Kendall’s coefficient of concordance in a voxel-wise level in three frequency bands so as to assess the similarity of the time series of its 26 nearest voxels (Zang et al., 2004). The standardized ReHo value was calculated as each subject’s ReHo value divided by the mean value of the entire brain. Finally, the ReHo maps for each participant were smoothed by a Gaussian filter of 4-mm full width at half maximum (FWHM) to reduce noise and residual differences and were used in statistical analysis (Guo et al., 2014).

### Statistical analysis

All statistical analyses were performed with Statistical Product and Service Solutions 26.0 (SPSS 26.0, IBM, Armonk, NY, United States) software. Categorical variables are presented as *n*, and continuous variables are presented as the mean ± standard deviation (*SD*). We performed a chi-square test to compare the gender difference between patients with BGIS and HCs. A two-sample *t*-test was performed to compare the age difference between the two groups. All tests of demographic information were two-tailed, and *p*-value < 0.05 was considered significant.

A two-sample *t*-test was performed to compare the ReHo maps between patients with BGIS and HCs. Frame-wise displacement (FD, Jenkinson) parameters (Jenkinson et al., 2002) and gender were regressed in the two-sample *t*-test to avoid the influence of head motion and differences in gender. A Gaussian Random Field (GRF; voxel *p* < 0.05, cluster *p* < 0.05, two-tailed) was conducted to get the brain regions with significant ReHo differences. To validate the stability of the results, the age and gender were also regressed in the statistical analysis, respectively, and the compared result maps after GRF correction (voxel *p* < 0.05, cluster *p* < 0.05, two-tailed) were provided (Supplementary Figures 1, 2). To support the sequential meta-analysis, we shared the original uncorrected *t* maps. (The original uncorrected *t* maps are available at http://www.restfmri.net/BGIS_ReHo.zip).

Pearson correlation analyses were conducted to assess the correlation between ReHo values and clinical scales (FMA-up, FMA-down, FMA, NIHSS, and BI).

## Results

### Participant’s characteristics

A total of 43 patients with BGIS and 47 HCs were enrolled in this study. Based on the exclusion criteria, 9 patients with BGIS and 3 HCs were excluded, leaving 34 patients with BGIS (25 men; mean age: 56.500 ± 10.999) and 44 HCs (19 men; mean age: 55.340 ± 11.485) listed in Table 1. No significant differences in age (*p* = 0.736) and education (*p* = 0.053) but in gender (*p* = 0.007), hypertension (*p* < 0.001), and diabetes (*p* = 0.041) were observed between HCs and BGIS group.

**TABLE 1:** Demographic and clinical characteristics of the participants.

Characteristic (*n*%)	BGIS (*n* = 34)	HCs (*n* = 44)	*P*-value
Age (years)	56.500 ± 10.999	55.340 ± 11.485	0.736
Gender			
Male	25 (73.5)	19 (43.2)	0.007**
Female	9 (26.5)	25 (56.8)	
Education (year)	11.500 ± 3.587	12.140 ± 3.008	0.053
Hypertension	26 (76.5)	7 (15.9)	<0.001***
Diabetes	9 (26.5)	4 (9.1)	0.041*
Lesion side			
Right	14 (41.2)	–	–
Left	20 (58.8)	–	–
Lesion			
Basal ganglia	1 (2.9)	–	–
Basal ganglia-capsula interna	4 (11.8)	–	–
Basal ganglia-corona radiata	20 (58.8)	–	–
Corona radiata	9 (26.5)	–	–
NIHSS score	3.760 ± 2.463	–	–
FMA score	74.910 ± 18.907	–	–
FMA-up	48.471 ± 14.028	–	–
FMA-low	26.441 ± 5.117	–	–
BI	75.441 ± 20.389	–	–

*p < 0.05; **p < 0.01; ***p < 0.001.

BGIS, Basal ganglia ischemic stroke; NIHSS, National Institutes of Health Stroke Scale; FMA, Fugl-Meyer Assessment scale; FMA-up, Fugl-Meyer Assessment scale of the upper limbs; FMA-low, Fugl-Meyer Assessment scale of the lower limbs; BI, Barthel Index.

### Regional homogeneity analysis in multifrequency bands

**FIGURE 1:**
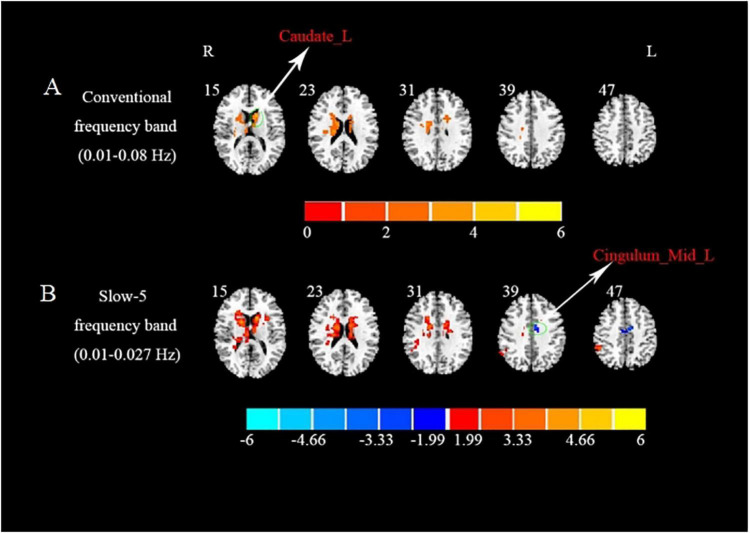
Brain regions with significant differences in ReHo in three different frequency bands between BGIS and HCs groups. **(A)** Brain regions with differences in ReHo in conventional frequency band. **(B)** Brain regions with differences in ReHo in slow-5 frequency band. R, right hemisphere; L, left hemisphere; BGIS, basal ganglia ischemic stroke; HCs, healthy controls; Caudate_L, left caudate; Cingulum_Mid_L, left medial cingulum.

In the conventional frequency band, compared with the HCs, significantly increased ReHo values were presented in the left caudate in the BGIS group (Table 2 and Figure 1A). We detected the alteration in some subregions of ReHo such as the right caudate and left putamen.

**TABLE 2:** Brain regions with significant differences in ReHo in three different frequency bands between ReHo in BGIS and HCs groups.

Brain regions (AAL)	MNI coordinates (X, Y, Z)	Cluster size	Peak *T*-value
**Conventional frequency band (0.01–0.08 Hz)**			
Caudate_L (aal)	(-12, 12, 15)	678	4.1215
Caudate_L (aal)		64	
Caudate_R (aal)		58	
Putamen_L (aal)		47	
**Slow-5 frequency band (0.01–0.027 Hz)**			
Cingulum_Mid_L (aal)	(-9, -6, 45)	1,446	-5.0606
Caudate_L (aal)		96	
Caudate_R (aal)		91	
Putamen_L (aal)		88	

The statistical threshold was set at voxel with p < 0.05 and cluster with p < 0.05 for multiple comparisons using Gaussian random field (GRF) theory corrected. In every cluster, the first label represents the brain region that peak voxel located, and the second label is the sub-region within the same cluster.

AAL, automated anatomical labeling; MNI, Montreal Neurological Institute; Caudate_L, left caudate; Caudate_R, right caudate; Putamen_L, left putamen; Cingulum_Mid_L, left medial cingulum.

In the slow-5 frequency band, compared with the HCs, the BGIS group showed a significantly decreased ReHo value in the left medial cingulum (Table 2 and Figure 1B). Some subregions with the alteration in ReHo such as bilateral caudate and left putamen were found in the BGIS group.

In the slow-4 frequency band, compared with the HCs, we did not find any differential brain regions in the BGIS group.

### Correlations analysis between regional homogeneity values and clinical scores

**FIGURE 2:**
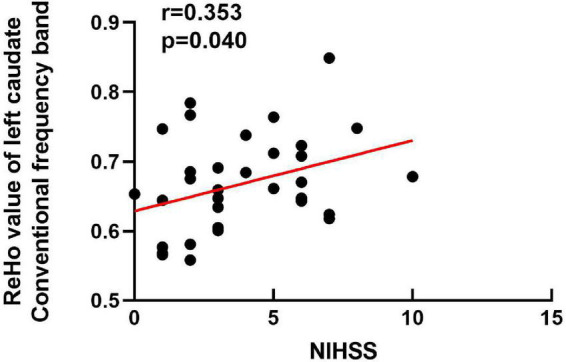
Correlation analysis between the NIHSS scores (x-axis) and ReHo (y-axis) in the left caudate of BGIS in conventional frequency band. NIHSS, National Institutes of Health Stroke Scale.

We explored the correlations between the ReHo values in these brain regions and clinical scores. Our results showed that the ReHo value of the left caudate of the BGIS group in the conventional frequency band was positively correlated with the NIHSS score (*r* = 0.353, *p* = 0.040) (Figure 2). It indicated that the higher the ReHo value, the higher the NIHSS score.

## Discussion

In this study, we utilized the ReHo analytical method in conventional frequency band, slow-5 frequency band, and slow-4 frequency band to investigate the alterations of intrinsic neural activities in patients with acute BGIS. Increased ReHo value was found in the left caudate of BGIS in the conventional frequency band while decreased ReHo value was found in the left medial cingulum of BGIS in the slow-5 frequency band. However, the decreased ReHo value in the left medial cingulum was only found in the slow-5 frequency band. The subregions cluster size of bilateral caudate and left putamen detected in the slow-5 frequency band were larger than those of the conventional frequency band. These findings indicated that ReHo alterations of BGIS were frequency-specific.

In this study, we found an increased ReHo value in the left caudate of the BGIS group in the conventional frequency band. A previous study demonstrated that caudate was one of the components of the BG region that played an important role in cognition, speech, and gait recovery after stroke (Grönholm et al., 2016; Lee et al., 2017; Zonneveld et al., 2019). Moreover, a diffusion tensor imaging (DTI) study focused on patients with chronic stroke reported that caudate volume ratio was positively correlated with motor function recovery (Yang et al., 2015). Li et al. (2021) showed that caudate was associated with fine motor disorders after stroke. These studies elucidated that there was a close relationship between the caudate and motor function after stroke. In accordance with these previous studies, our findings may suggest that the enhanced synchronicity of neural activities of caudate in the BGIS group may be related to motor function after stroke. A previous study indicated that transcranial magnetic stimulation (TMS) on the caudate could enhance motor cortex excitability, which may be helpful for motor function recovery (Bestmann et al., 2005). Hence, we speculated that the caudate might be taken as the stimulus targeted point in rehabilitation therapy, which may be useful in motor functional recovery. But we failed to find any relationship between caudate and motor dysfunction in patients with BGIS. More trials that caudate work as a rehabilitation target after stroke are still required for the validation of this point in the future.

Our results showed that compared to the HCs, ReHo changed in the left putamen of the BGIS group in the conventional frequency band. Putamen as a nucleus of the BG was located near the motor never tracts of the brain (Frenkel-Toledo et al., 2022). Damage to the corticospinal tract in the putamen would affect lower limb motor function of patients with stroke (Frenkel-Toledo et al., 2021). And ischemic impairment of the putamen was found to be associated with the motor dysfunction in animal ischemic stroke models (Scheulin et al., 2021). In addition, a previous study showed that the low variability of dynamic connectivity between bilateral putamen and the supplementary motor area was related to a better motor recovery of patients with stroke (Bonkhoff et al., 2021). Based on the evidence above, the abnormal neural activity of the putamen in our study may imply motor function impairment after BGIS.

In this study, our results showed that the BGIS group exhibited decreased ReHo value in the left medial cingulum only in the slow-5 frequency band compared with HCs. A previous study revealed that injury to the cingulum would disrupt the function of the medial motor system resulting in underutilization of contralesional limbs in patients with stroke (Migliaccio et al., 2014). A DTI study revealed that repetitive transcranial magnetic stimulation (rTMS) on patients with stroke could promote reorganization of the cingulum to improve the recovery of limb motor function (Li et al., 2018). It indicated that the cingulum was related to motor dysfunction after stroke. Moreover, the abnormal neural activity of the anterior cingulum caused by stroke was associated with the damage to white matter, and it may cause deficit in cognition (Pirondini et al., 2022). Hence, the alteration of ReHo in the cingulum suggested neural function impairment after BGIS.

In sub-frequency bands, frequency-specific properties of ReHo have been found in our study. Specifically, we noticed that the subregions cluster size of bilateral caudate and left putamen detected in the slow-5 frequency band were larger than those of the conventional frequency band. And the decreased ReHo value of the left medial cingulum was only detected in the slow-5 frequency band. Hence, it indicated that the slow-5 frequency band was more sensitive in detecting the abnormal neuronal activity of patients with BGIS, and ReHo analysis of the slow-5 frequency band was more helpful to detect an extensive neural activity of BGIS. Our previous study also suggested the frequency-specificity of slow-5 (Quan et al., 2022). Zhu et al. (2015) suggested that the slow-5 frequency band was more sensitive to the alteration of ReHo of stroke. This property of the slow-5 frequency band is also reported in functional connectivity (Li et al., 2022). In addition, our findings were consistent with previous results that the slow-5 frequency band was better at reflecting gray-matter LFO signals and widespread cortical intrinsic neural activities (Zuo et al., 2010; Zhu et al., 2015). Therefore, our study demonstrated the frequency-specific properties of BGIS. In future fMRI studies of BGIS, we should take frequency-specific properties into account.

We found a positive correlation between the ReHo value of the left caudate and NIHSS score in conventional frequency band, which means that as the ReHo value increases, the NIHSS score of the patient with BGIS is higher. The caudate was one of the BG nuclei, which was involved in motor control (Lehéricy and Gerardin, 2002). Moreover, caudate was also found to be involved in language control, and abnormal caudate neural activity may lead to speech dysfunction in patients with stroke (Crinion et al., 2006). The NIHSS score can assess the severity of stroke, which includes speech and motor function. A study showed that disruption in functional networks of the caudate may be associated with cognitive impairment in patients with stroke (Miao et al., 2021). The alteration of neural activity in the caudate may indicate motor, speech, and cognitive impairment in the patient with BGIS. Therefore, the ReHo value of the left caudate in the conventional frequency band may work as a marker to assess the neural impairment caused by BGIS.

In this study, results showed that some of the clusters are located in the CSF. The BOLD signal of CSF and white matter (WH) was traditionally regarded as physiological noise in a previous fMRI study (Bartoò et al., 2019). However, in recent years, studies have shown that CSF and WH BOLD signal could also reflect neural activity (D’Arcy et al., 2006; Gawryluk et al., 2011). A fMRI study of Parkinson’s disease (PD) showed a CSF signal, which may be helpful for the early diagnosis of PD (Long et al., 2012). Besides, a study of psychosis highlighted the importance of CSF BOLD signals (Saarinen et al., 2020), and the CSF pulse of BOLD could be used for further pathological analysis of neural disease (Kim et al., 2022). Hence, the CSF BOLD signal was significant in reflecting neural activity. The necessity of CSF signal as physiological noise correction was still under discussion (Birn, 2012). Although our results displayed alteration, ReHo of CSF region and the meaning of CSF region needed further study.

### Limitations of the study

There are several limitations to our study. First, because of the strict inclusion and exclusion criteria, the sample size of our study is relatively small. Second, there are large gender differences between BGIS and HCs groups due to the strict inclusion criteria. Third, the lateral of lesions was not differentiated in this study, and we could not rule out the influence of lateralization. Fourth, although we have performed additional statistical analyses to validate the stability of the results of this study, the findings that patients with BGIS and HCs showed significant differences in CSF regions should be explored in the future studies with a larger sample size.

## Conclusion

The findings of this study revealed the abnormal local synchronization of intrinsic neural activities among patients with acute BGIS in three different frequency bands and helped us to understand the neural mechanisms of BGIS. Importantly, we found the alteration of ReHo of BGIS was frequency-specific and suggested that the use of a specific frequency could detect more extensive alterations of ReHo in BGIS. Future BGIS fMRI studies should consider the frequency-specific effects.

## Data availability statement

The raw data supporting the conclusions of this article will be made available by the authors, without undue reservation.

## Ethics statement

The studies involving human participants were reviewed and approved by the Ethics Committee of First Affiliated Hospital, Guangxi Medical University. The patients/participants provided their written informed consent to participate in this study.

## Author contributions

ZL, XJ, and QL conceived the study. HC, YM, XQ, and CM collected the materials and performed the statistical analysis. QL and SH wrote the first manuscript. SH analyzed the fMRI data. ML and LZ revised the manuscript. YG, ZH, and LC helped coordinate the study and reviewed the manuscript. All authors read and approved the present text.

## Footnotes

1
https://uk.mathworks.com/products/matlab

2
http://restfmri.net/forum/restplus

3
http://www.fil.ion.ucl.ac.uk/spm

4
http://rfmri.org/dpabi
