# Supplementary material for: Regional homogeneity alterations in multifrequency bands in patients with basal ganglia stroke: A resting-state functional magnetic resonance imaging study
Source: Front Aging Neurosci. 2022 Aug 11;14:938646. doi: 10.3389/fnagi.2022.938646 (PMC9403766; doi:10.3389/fnagi.2022.938646)
Supplement: Supplementary file 1 [file Data_Sheet_1.PDF]

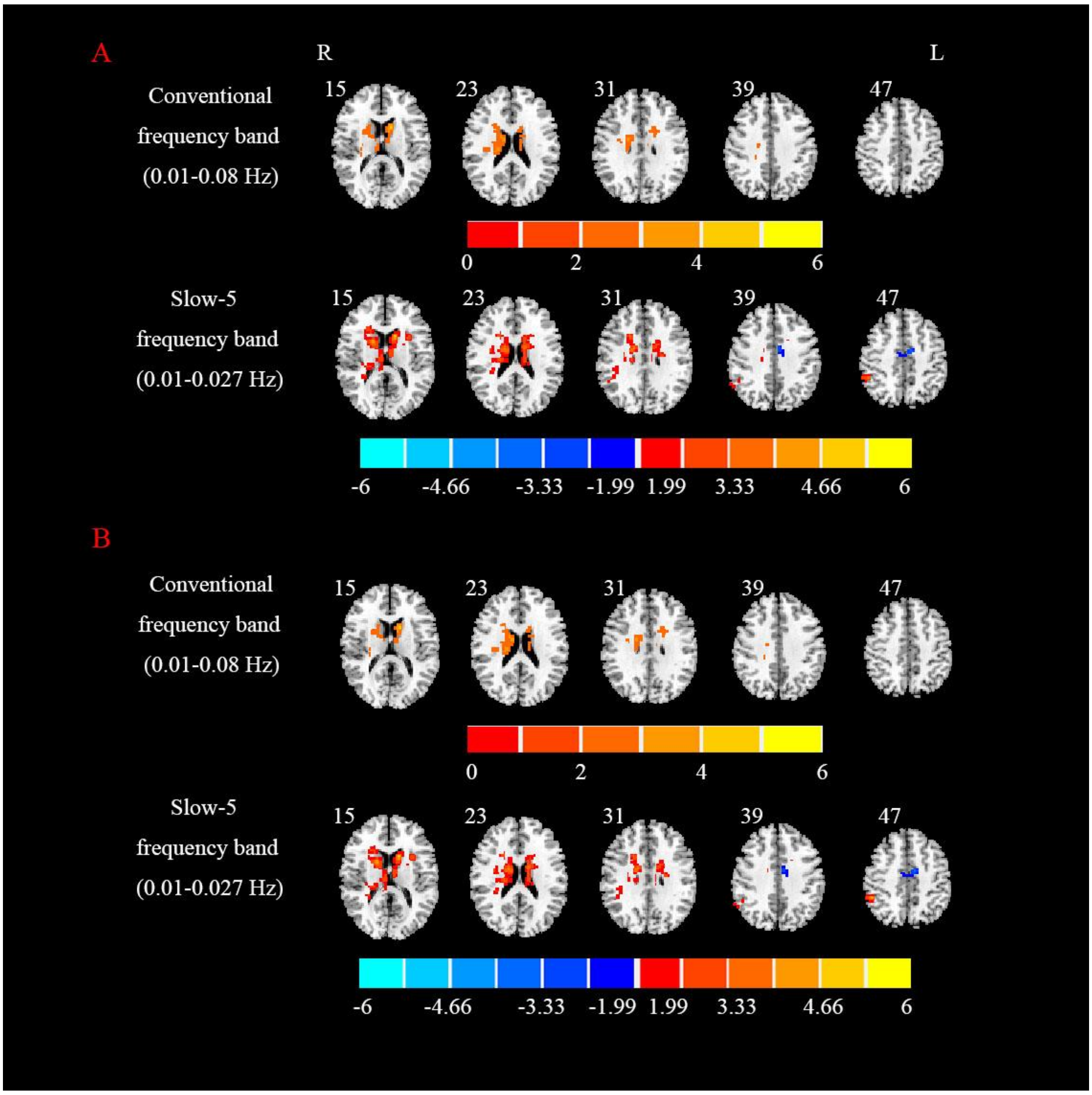

Figure S1. The compared pattern map before and after adding age as covariable.

Note: The statistical threshold was set at voxel with $p<0.05$ and cluster with $p<0.05$ for multiple comparisons using Gaussian random field(GRF) theory corrected. In the figure, A represents the result before regression of age, and B represents the result after regression of age.

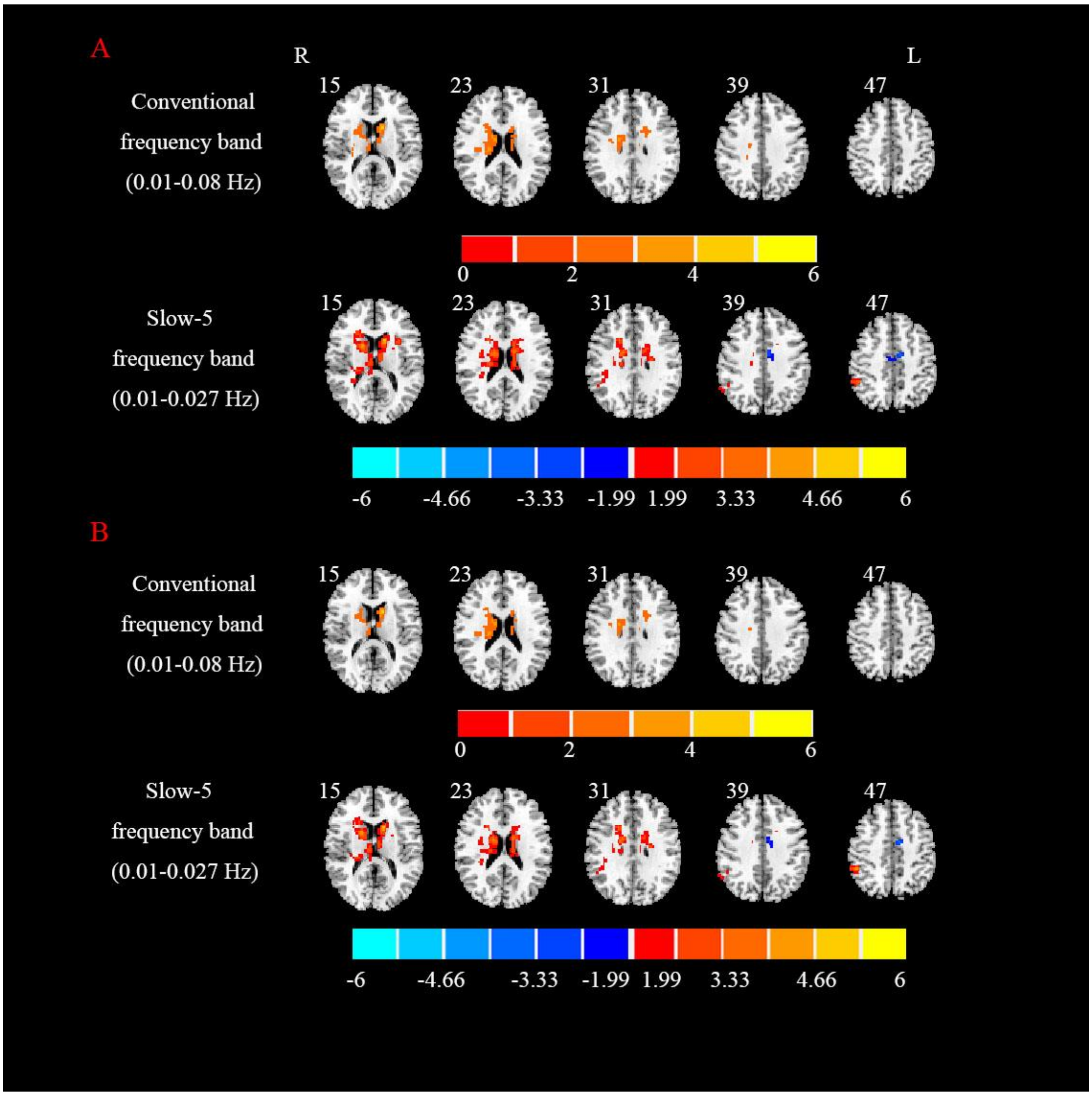

Figure S2. The spatial comparison patterns before and after adding education level as covariable.

Note: The statistical threshold was set at voxel with $p<0.05$ and cluster with $p<0.05$ for multiple comparisons using Gaussian random field(GRF) theory corrected. In the figure, A represents the result before regression of education level, and B represents the result after regression of education level.
